# Acute Effects of Oatmeal on Exercise-Induced Reactive Oxygen Species Production Following High-Intensity Interval Training in Women: A Randomized Controlled Trial

**DOI:** 10.3390/antiox10010003

**Published:** 2020-12-22

**Authors:** Zhen Zeng, Patrick Jendricke, Christoph Centner, Helen Storck, Albert Gollhofer, Daniel König

**Affiliations:** 1 Department of Sport and Sport Science, University of Freiburg, 79098 Freiburg, Germany; patrick.jendricke@sport.uni-freiburg.de (P.J.); christoph.centner@sport.uni-freiburg.de (C.C.); helen.storck@neptun.uni-freiburg.de (H.S.); ag@sport.uni-freiburg.de (A.G.); daniel.koenig@sport.uni-freiburg.de (D.K.); 2 School of Sports Medicine and Health, Chengdu Sport University, Chengdu 610000, China; 3 Praxisklinik Rennbahn, 4132 Muttenz, Switzerland; 4 Department of Sports Science, Institute for Nutrition, Sports and Health, University of Vienna, 1090 Vienna, Austria

**Keywords:** oatmeal, high-intensity interval training, reactive oxygen species, pre-exercise meal

## Abstract

High-intensity interval training (HIIT) has been demonstrated to increase the generation of reactive oxygen species (ROS). Therefore, strategies to mitigate excessive ROS productions could be useful to reduce the negative consequences of oxidative damage for health, as well as for physical, performances. The aim of this study was to investigate the acute effects of pre-exercise oatmeal consumption on exercise-induced ROS generation in young, healthy women. Thirty-four participants were randomly allocated in one of two groups: oatmeal prior to HIIT (oatmeal; *n* = 17) or HIIT alone (control; *n* = 17). Blood samples were obtained at pre-meal, pre-HIIT, immediately post-HIIT, and 15 min after HIIT. Electron paramagnetic resonance (EPR) spectroscopy was used to analyze the concentrations of ROS in the capillary blood. In addition, the blood glucose and blood lactate levels were measured. Immediately post-HIIT, the ROS generation in the oatmeal group was significantly lower in contrast to the control group (*p* < 0.05). A significant interaction effect of time × meal (*p* < 0.05; η^2^ = 0.234) was detected from the pre-meal to 15 post-HIIT for ROS production. Moreover, significant differences in the blood glucose levels were observed between the groups at pre-HIIT and immediately post-HIIT (*p* < 0.05). In conclusion, the consumption of oatmeal before HIIT may mitigate exercise-induced ROS production.

## 1. Introduction

In recent years, the effects of exercise on the generation of reactive oxygen species (ROS) have been extensively investigated [1]. Previous research indicated that the physiological levels of ROS produced during moderate-intensity exercise are essential for optimal cell function and signal transduction [2,3]. However, exercising with strenuous and vigorous intensity can augment ROS generation and, thus, may lead to an overload of the antioxidant capacity, with a subsequent induction of oxidative stress [1,4,5]. The excessive ROS produced during fatiguing exercise has been described to be involved in the development of muscle damage [6,7] and is associated with aging [8,9] and with chronic degenerative diseases [10]. The low amounts of ROS generated intermittently during a regular training protocol program can activate intracellular signaling ways (e.g., nuclear factor-κB (NF-κB), nuclear factor erythroid 2-related factor 2 (NRF2) and mitogen-activated protein kinase (MAPK)) that promote cellular adaptations, leading to increased capacities against subsequent stresses [11,12]. One of the potential mechanisms underlying this adaptation is that the cells exposed to ROS are brought to respond by inducing or repressing a remarkable variety of target genes [13]. The important reason for the ability of redox signaling to alter gene expression and modify the muscle phenotype includes changes in the transcriptional activator phosphorylation status due to the ability of ROS to regulate the activities of several kinases and phosphatases [11]. Thus, in contrast to regular low-intensity training, a single bout of high-intensity exercise leads to oxidative stress attributed to the lack of adaptations that can upregulate the antioxidant defense system. Therefore, the research is challenged to explore interventions capable of protecting the human body from exercise-induced oxidative stress, both under resting conditions, as well as during exercise.

In terms of potential countermeasures, previous studies have reported the beneficial effects of antioxidants such as vitamin C [14], vitamin E [15], Se [16], and the combination of these supplements [17] on inhibiting exercise-induced oxidative stress.

Apart from specific substances, recent investigations have revealed that certain meal compositions seem to impact the level of oxidative stress agents following exercise. Especially, avenanthramide (AVA), which is mainly found in oats, has been a well-discussed topic in the scientific community due to its strong antioxidant activity in animal and human studies [18,19,20,21]. Previous studies showed that both the hydroxyl groups and the α, β-unsaturated carbonyl moiety are crucial for the antioxidant properties of AVAs, and they act directly or indirectly to scavenge ROS [22].

Koenig et al. [18] demonstrated that long-term oat AVA supplementation can attenuate blood inflammation markers, decrease ROS generation and mononuclear cell NF-κB activation, and increase the antioxidant capacity during an eccentric exercise bout in women. Likewise, chronic AVA supplementation can diminish ROS generation, as well as peripheral inflammatory and immunological markers, in response to an acute bout of downhill running [19]. However, these investigations have only used indirect measurements of ROS generation. Indirect techniques for oxidative stress quantification are based on the determination of end products of the oxidative damage resulting from the interaction of ROS with biological macromolecules rather than a direct measurement of the ROS generation [23]. Moreover, no study has determined the effects of AVA ingestion prior to a bout of high-intensity interval training (HIIT) on the acute production of ROS so far. This research will provide clinical evidence for effectiveness of pre-exercise diets as a practical strategy for reducing the magnitude of exercise-induced ROS production.

Accordingly, the main objective of this study was to examine the acute effects of an oatmeal on exercise-induced ROS production in women. In order to elicit a sufficient stimulus for ROS production, a HIIT was implemented. With the use of the electron paramagnetic resonance (EPR) technique, we are, for the first time, able to directly quantify ROS [24,25] instead of the indirect assessments that were previously used. As a secondary outcome, changes in the blood glucose and lactate values were analyzed in order to quantify the metabolic response to exercise.

## 2. Materials and Methods

### 2.1. Subjects

In total, thirty-four healthy female subjects with a body mass index (BMI) between 18.5 and 26 kg∙m^−2^ and a body fat percentage of >20% were included in this study. Participants were not eligible if they had any contraindications with regards to physical activity according to American College of Sports Medicine (ACSM) guidelines, including cardiovascular, metabolic, or renal diseases [26]. The regular physical activity level of each participant did not exceed 120 min per week, as assessed by the Freiburg questionnaire of physical activity [27]. Further exclusion criteria were smoking, pregnancy, and medications affecting plasma ROS concentrations. All participants were advised not to take antioxidant or anti-inflammatory supplements during this experiment. In order to avoid interference from hormonal changes [28], no subjects underwent the measurements during their menstrual periods.

The study was approved by the local ethics committee and carried out according to the latest revision of the Declaration of Helsinki. Experimental procedures and potential risks were explained before informed consent was obtained prior to inclusion. The trial was registered with DRKS-ID: DRKS00023749. Ethics Committee of the University of Freiburg (ETK: 43/18). The date of Ethics Committee Application is: 16. April. 2018. The date of Ethics Committee Approval is: 28. August. 2018. For measurement of ROS in this study, we wrote an amendment with the respective design and methodological modifications which was approved on 4. July. 2019 (ETK: 43/18-190309)

### 2.2. Study Design

The present study was designed as a repeated-measures, randomized, single-blind trial conducted at the University of Freiburg in Freiburg, Germany. For this purpose, randomization was performed using a random number generator [29]. All investigators were blinded until all the data was entered and the statistical analysis was performed.

**Figure 1:**
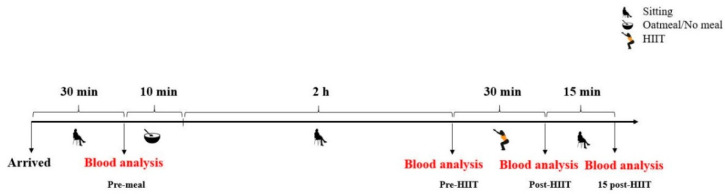
Study design overview. HIIT: high-intensity interval training.

**Figure 2:**
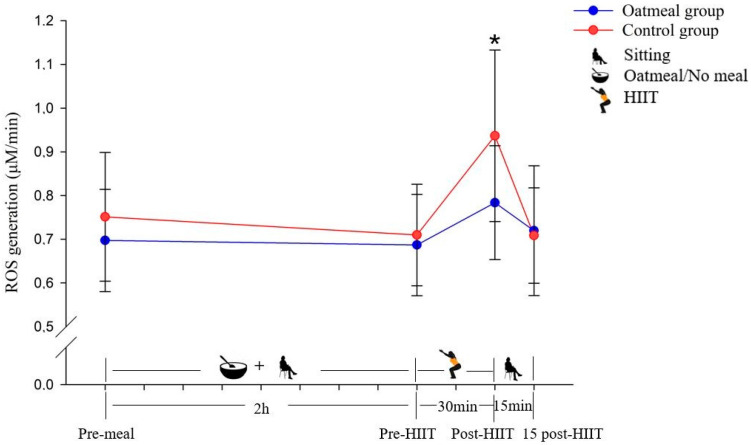
Changes of the reactive oxygen species (ROS) generation in μM/min (mean ± SD) from pre-meal to 15 post-HIIT in the two groups (* *p* < 0.05; a significant difference between the oatmeal group and control group was detected by an independent *t*-test).

**Figure 3:**
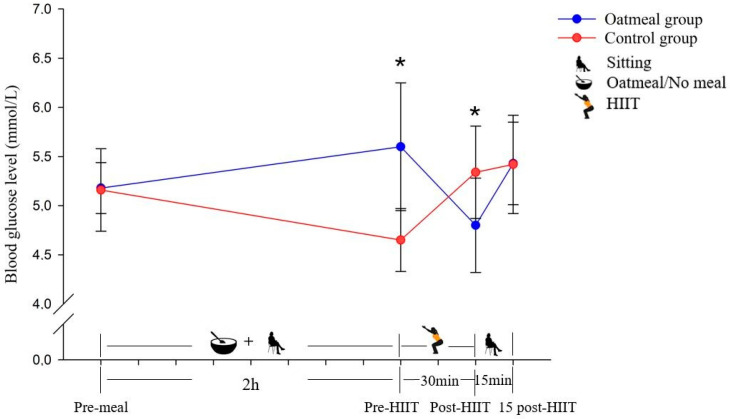
Changes of the blood glucose level in mmol/L (mean ± SD) from pre-meal to 15 post-HIIT in the two groups (* *p* < 0.05; significant differences between the oatmeal group and control group were detected by an independent *t*-test).

Prior to the experiment, all participants were randomly allocated into one of the following two groups: the consumption of oatmeal followed by HIIT (oatmeal; *n* = 17) and HIIT only (control; *n* = 17). Experiments began at 8 a.m. after a 12-h fasting period. In order to reduce and ensure stable measuring conditions for ROS production, all participants were required to sit and rest for 30 min before the first measurements and during the resting period of the experiment. After the consumption of oatmeal or no meal (10 min), the participants remained seated for two hours. Subsequently, after performing the training intervention, they continued to rest in their seats for 15 min. Total ROS generation, as well as glucose and lactate concentrations, were analyzed from the capillary blood prior to and 120 min after consumption of the test meal. Additionally, these markers were again measured immediately and 15 min after the exercise session. These four measurements are designated as pre-meal, pre-HIIT, post-HIIT, and 15 post-HIIT, respectively, in Figure 1, Figure 2, and Figure 3.

### 2.3. High-Intensity Interval Training (HIIT)

HIIT involves alternating short bursts of high-intensity exercise with recovery periods or light exercise [30]. Based on the study from Klika et al. [31], subjects conducted a high-intensity interval training for the lower limbs, performing 3 sets squats, lunges, and one-legged heel rises using each individual’s bodyweight as resistance. Participants were encouraged to maintain an execution speed of 2 s, which was equally split between the concentric and eccentric phases. A 30-s rest interval was provided between sets [32,33].

### 2.4. Oatmeal

The oatmeal was consumed by the oatmeal group after the first blood drawing. This meal contained 1g∙kg∙body mass (BM)^−1^ of carbohydrates and consisted of oat flakes (© Peter Kölln GmbH & Co. KGaA, Elmshorn, Germany) and semi-skimmed milk (1.5% fat). Table 1 shows the nutritional composition of the oatmeal. Additionally, each participant consumed a standardized volume of fluid (650 mL of water and milk) with their meal [34].

Since ROS production, the lactate concentration, and glucose levels can be influenced by dietary intake, the participants were instructed to abstain from alcohol 48 h before the measurements and arrived at the investigation site following a 12-h overnight fasting.

### 2.5. Blood Analysis

The total ROS formation rate was measured using the electron paramagnetic resonance (EPR) technique (Bruker, Germany) equipped with a temperature and gas controller BIO-III (TGC-BIO, Noxygen Science Transfer & Diagnostics GmbH, Elzach, Germany) to ensure analyses under in vivo conditions at 37 °C. For each sample test, 10-μL freshly drawn capillary blood from each participant was sampled using a standard Eppendorf pipette (Eppendorf, Research Plus, Hamburg, Germany). In order to monitor the cellular oxygen consumption, we added 10 μL of oxygen-sensitive label (Noxygen, catalog number: NOX 15.1–5 μmol/L) [37] to the 10 μL blood sample, then vortexed the mixture. Subsequently, 20 μL of Krebs-HEPES buffer-diluted spin probe 1-hydroxy-4-phosphonooxy-2,2,6,6-tetramethylpiperidine (CMH, 400 μmol/L) was mixed into the 20-μL blood-label solution. The oxygen-sensitive label and the CMH were obtained from Noxygen Science Transfer & Diagnostics (Elzach, Germany). In this condition, released ROS in the capillary blood interacts in the intracellular and extracellular space with CMH to form the stable radical CM• [38]. After transferring the 40μL mixture into a capillary tube (Hirschmann, Eberstadt, Germany), the ROS formation rate was measured using the following settings: center field: g = 2.011, sweep width: 60 G, sweep time: 5.24 s, frequency: 9.76 GHz, power: 20 mW, gain: 1 × 10^3^, modulation amplitude: 1.0 G, and number of scans: 10. Calibration of the EPR signal was performed using a standard 10 μM solution.

To measure the lactate and glucose concentrations, 20 μL of capillary blood was obtained from the right index finger immediately after the ROS sample was obtained. Subsequently, the blood-filled plastic capillary tube was placed in a 1000 μL safe-lock tube with a hemolysis solution (Eppendorf Tubes^®^, Eppendorf, Germany). This sample mixture was analyzed by an enzymatic-amperometric method using a Biosen S-Line Lab plus lactate and glucose analyzer (EKF Diagnostics, Cardiff, UK).

### 2.6. Body Composition

The body fat percentage and body mass index (BMI) of participants were measured in their fasting state. The body analysis monitor OMRON BF500 (OMRON, Medizintechnik Handelsgesellschaft GmbH, Mannheim, Germany) was used for measurements by means of electrodes on the extremities using a bioelectrical impedance analysis (BIA). In conformity with the guidelines of the European Society for Clinical Nutrition and Metabolism (ESPEN) [39], BIA assessments were accomplished. All measurements were standardized performed after an overnight fast of 12 h in the morning between 8 a.m. and 11 a.m. Before every measurement, the participants were asked to abstain from physical exercise and alcohol for at least 48 h.

### 2.7. Borg Scale of Perceived Exertion

Borg’s rating of perceived exertion (RPE) was used to assess the subjective perception of effort during the training intervention in this study [40]. Each participant was required to indicate their Borg Scale at the pre-meal, pre-HIIT, post-HIIT, and 15 post-HIIT time points.

### 2.8. Statistical Analysis

SPSS Statistics software version 24.0 (IBM, Armonk, NY, USA) was used for all statistical analyses, using an alpha level of *p* < 0.05. After checking for the normal distribution of all variables by using the Kolmogorov-Smirnov test, independent *t*-tests (between two groups) were performed for the examination of significant differences. Interaction effects were tested with a four (time) × two (condition) repeated measures ANOVA (RMANOVA). An outlier analysis (mean ± 2 standard deviation (SD)) was performed based on the ROS results, and *n* = 2 subjects (one for the oatmeal group and one for the control group) were excluded from the analysis. The statistical analysis of all the data was completed after the removal of these two outliers. All data are presented as the mean ±SDin the figures and tables.

## 3. Results

All 34 participants completed the trial, and no adverse events were noted. The participant characteristics are presented in Table 2. However, due to an outlier analysis, two subjects were not included in the data analysis.

### 3.1. Reactive Oxygen Species Production

The ROS production was significantly different between the oatmeal group and the control group immediately following the HIIT exercise (*p* < 0.05) (Figure 2). No statistically significant differences were detected at the pre-meal, pre-HIIT, and 15-post HIIT time points. Following the calculation of the RMANOVA, the significant interaction effect of time × meal (*p* < 0.05; η^2^ = 0.234; Table 3), and main effect of time (*p* < 0.05; η^2^ = 0. 524) were detected with a greater increase in ROS from pre-meal to 15 post-HIIT compared to the control. In contrast, it did not show a significant main effect of the condition (*p* = 0.087; η^2^ = 0.194).

### 3.2. Glucose

The glucose levels of the oatmeal group significantly increased at the pre-HIIT time point (*p* < 0.05) and decreased significantly at the post-HIIT time point (*p* < 0.05) compared with the control group (Figure 3). From pre-meal to 15 post-HIIT, the analysis of the RMANOVA of glucose demonstrated a significant main effect of the time (*p* < 0.05; η^2^ = 0.332) and time × condition interaction (*p* < 0.05; η^2^ = 0.686; Table 3). In contrast, it did not show a significant meal condition effect (*p* = 0.379; η^2^ = 0.056).

### 3.3. Blood Lactate

No difference in the blood lactate levels could be detected between the two groups at each time point. From pre-meal to 15 post-HIIT, the analysis of RMANOVA showed a significant main effect of the time (*p* < 0.05; η^2^ = 0.787) but did not show a significant main effect of the meal condition (*p* = 0.478; η^2^ = 0.037) or time × meal condition interaction (*p* = 0.600; η^2^ = 0.020; Table 3).

### 3.4. Borg Scale

Table 4 described that there is no significant difference of the Borg Scale between two groups at each time point. The results of the RMANOVA analysis revealed a significant main effect of time (*p* < 0.05; η^2^ = 0.950) from pre-meal to 15 post-HIIT, but there was no significant main effect of the meal condition (*p* = 0.097; η^2^ = 0.185) and time × meal interaction (*p* = 0.987; η^2^ = 0.003).

## 4. Discussion

The main finding of this study was that a pre-exercise oatmeal consumption reduced exercise-induced ROS production after HIIT. The significant main effects of time × meal condition and time were detected from pre-meal to 15 post-HIIT. Additionally, the oatmeal intake significantly increased the postprandial blood glucose levels after two hours and significantly decreased the blood glucose levels after training compared to the control group. To the best of our knowledge, this study was the first to examine the acute effects of oatmeal on exercise-induced ROS production following HIIT.

In the present study, oat flakes and semi-skimmed milk were used as the test meal. In terms of a food component, the potential antioxidant capacity of the oats (*Avena sativa* L.) [41] and milk [42] were already shown.

Avenanthramides (AVAs), as a group of phenolic compounds, were considered the most important antioxidants in the oat flakes [43,44]. There are more than 20 different forms of AVA present when extracted from oats. AVA-C, one of the three major forms, was shown to have the highest antioxidant activity in vitro [43]. The concentration in groats of these three AVAs ranged between 2.5–4.7mg/100 g (AVA-A), 2.1–4.3 mg/100g (AVA-B), and 2.8–6.2 mg/100 g (AVA-C) [45]. The antioxidant activity of AVA is 10–30 times greater than that of the oats’ other phenolic antioxidants, such as vanillin and caffeic acid [46]. Although the strong antioxidant capacity of AVAs has been demonstrated in in vivo and in vitro studies, the underlying mechanisms for these effects are largely unknown. An in vitro study evaluated the hydroxyl groups of AVAs that are involved in free radical trapping, which accounts for the ability of AVAs to combat oxidative stress [47]. Another recent study suggested that AVAs are able to activate the NRF2 defense system against oxidative stress that involves the α, β-unsaturated carbonyl moiety [48]. The α, β-unsaturated carbonyl groups of AVAs act as Michael acceptors for a nucleophilic attack [22]. Thus, their hypothesis was proved by their observation that AVAs significantly increase the heme oxygenase-1 (HO-1) expression in human kidney-2 (HK-2) cells, and this upregulation of HO-1 expression is mediated by NRF2 translocation and ROS [48]. However, these still need further clarification.

Previous research findings have indicated AVA’s antioxidant and anti-inflammatory properties in both preclinical and human studies [18,19,20,21]. Zhang et al. [19] and Koenig et al. [18] investigated the effects of chronic AVA supplementation on exercised-induced ROS generation in their clinical studies. Koenig et al. [18] demonstrated in their study that participants supplemented with two AVA-enriched cookies daily for eight weeks decreased the ROS generation from the neutrophil respiratory burst (NRB) and plasma C-reactive protein (CRP) levels after a high-intensity training. In a further investigation with the same intervention, Zhang et al. [19] proved that NRB played a key role in ROS generation, and AVA can reduce this source of ROS generated from neutrophils (or monocytes) catalyzed by nicotinamide adenine dinucleotide phosphate (NADPH) oxidase. These studies confirmed the AVA’s antioxidant and anti-inflammatory effects on exercise-induced oxidative stress by using indirect measuring methods. Thus, the changes in the ROS generation were interpreted only through NRB and plasma CRP levels or the protein expression, whereas the present study applied direct ROS measurements using EPR technology.

Meanwhile, oat contains the following trace amounts of antioxidant vitamins: vitamin A: 0.862 mg/100 g (mucilage), β-carotene: <1.0 μg/100 g (rolled oat), and vitamin E (total): 0.2–0.8mg/100 g [49,50]. Besides, phytic acid, flavonoids, sterols [41], and the dietary fiber beta-glucan [51] are also presented as antioxidants in oats. However, they are all trace ingredients.

Additionally, the lipophilic antioxidants (conjugated linoleic acids, vitamins A and D3, vitamin E, ß-carotene, coenzyme Q10, and phospholipids), as well as the hydrophilic antioxidants (proteins, peptides, vitamins, minerals, and trace elements) are all discussed to play a role in the antioxidant effects of milk [42,52]. Lipophilic and hydrophilic antioxidants interact in the process of deactivating ROS and the finial products of lipid peroxidation [52].

In our study, no hypoglycemia (<3.5 mmol/L) was observed in any of the groups, and there were no differences in the blood lactate and RPE between the groups. The Borg Scale ratings of the two groups (oatmeal = 16.0 ± 1.4 and control = 15.3 ± 2.3) showed that all the participants reached the level of maximal exhaustion [40,53]. In intense exercise in people without diabetes, although the fluctuation of blood glucose is influenced by the pre-exercise diet, the plasma insulin levels can correct the glucose level and restore muscle glycogen [54]. In the oatmeal group, the blood glucose increased after consuming the oatmeal because of the food’s effects. Afterwards, it decreased after the HIIT session, since the oatmeal, which can be defined as a low-glycemic index food, might have reduced the blood glucose responses during training. Conversely, the blood glucose in the control group showed an opposite trend, which was attributed to the lower pre-exercise glucose and the reactive hyperglycemia after training. However, at the 15-post HIIT time point, the blood glucose of both groups returned to the same level. Furthermore, no difference in blood lactate levels could be detected between the two groups at each time point, which indicated that there was no significant difference of exercise intensity between the two groups.

Regarding the dietary strategies to avoid oxidative stress caused by exercise and the subsequent muscle damage, McAnulty et al. reported that the administration of 150-g/d blueberries, rich sources of polyphenols, could significantly decrease the endurance running-induced overproduction of free radicals in athletes in a hot environment [55]. In another study of elite male athletes, researchers demonstrated that the ingestion of 400mg of grape extract per day during one month resulted in a 9.48% increase in antioxidant capacity determined by the oxygen radical absorbance capacity (ORAC) method compared to placebo ingestion [56]. Chang et al. demonstrated that consuming a high-polyphenol diet (purple sweet potato leaves) for seven days could modulate the antioxidative status and decrease one h of treadmill running-induced oxidative damage [57]. In summary, blueberries, grapes, and purple sweet potato leaves are all rich in polyphenols, which played crucial antioxidant roles in these studies. Polyphenolic phytochemicals, including AVA, have been shown to have an antioxidant capacity in exercise-induced oxidative stress [46].

Nevertheless, the present study has some limitations. In this investigation, only young and premenopausal women were included. Considering the potential gender-related differences in oxidative stress, only female subjects were enrolled in this study. Although evidence for this hypothetical theory remains scarce, some previous human studies have reported that females have lower oxidative stress levels at all time points when compared to equally trained males [58,59]. The greater resistance against oxidative stress of female can be explained by the antioxidant effect of estrogen [60]. Strelow et al. demonstrated that estrogen acts as an antioxidative via the stimulation of manganese superoxide dismutase (MnSOD) and extracellular superoxide dismutase (ecSOD) expression and activity in circulating monocytes [60]. Furthermore, no subjects underwent the measurements during their menstrual periods in order to avoid disruptions caused by hormonal changes [28]. Since ROS production is also influenced by numerous factors, including training status, sex, and age, our results are not necessarily applicable to other populations. A previous study proposed that the oxidative stress response of an individual is highly variable due to the individual nature of redox control [61]. In order to quantify this system, although EPR spectroscopy is the only direct measure of free radicals, future studies should consider combining direct and indirect methods to achieve more precise results [62]. Moreover, the exact mechanisms of how the oatmeal reduced ROS production are not clear. Since the present study aimed to investigate the acute effects of an oatmeal on exercise-induced oxidative stress, further studies are needed that focus on the antioxidant capacity of this meal composition concomitant with a placebo meal group. Additionally, the exact fiber-type composition was not available from the manufacturers, which limited the conclusions about the potential effects of specific ingredients on ROS production.

## 5. Practical Application

In the field of sport and exercise science, carbohydrate ingestion prior to exercise has been proven to affect the metabolic responses and performance [63,64]. Oatmeal with a low glycemic index was proved to be associated with an increased availability of glucose to the working muscles [34]. The results of this study suggest that pre-exercise oatmeal consumption containing high amounts of AVAs might be considered as a measure to reduce high-intensity exercise-induced oxidative stress. Future studies should investigate if a diet containing reasonable amounts of oats with antioxidant potential might be used to prevent exercise-induced muscle fatigue, reduce exercise-induced muscle damage, and, subsequently, accelerate post-exercise recovery.

## 6. Conclusions

In conclusion, the consumption of a pre-exercise oatmeal resulted in a statistically significant reduction in postprandial exercise-induced ROS generation in women. Further studies with a longitudinal study design are warranted in order to investigate the chronic effects of oatmeal on exercise-induced ROS production.

## Figures and Tables

**Table 1:** Nutritional composition of the oatmeal.

TitleNutrients	Oat (per 100 g) [35]	Skim Milk (per 100 mL) [36]
Energy (kcal)	361	48
Carbohydrate (g)	56	5.0
Fat (g)	6.7	1.5
Protein (g)	14	3.5
Unsaturated fatty acids (g)	5.4	0.5
Saturated fatty acids (g)	1.3	1.0
Sugar (g)	1.2	5.0
Fiber (g)	11	0

**Table 2:** Subject descriptive and anthropometric characteristics (*n* = 32).

Variable	Mean ± SD	MIN	MAX
Age (years)	25.5 ± 5.1	21	37
Height (cm)	167.4 ± 6.4	156	180
Weight (kg)	62.5 ± 6.1	54.2	73.8
BMI (kg·m^−2^)	22.3 ± 1.9	18.7	25.5
Body fat (%)	31.4 ± 3.8	21.8	39.2

BMI: body mass index.

**Table 3:** Changes in the metabolic parameters in the oatmeal group and control group from pre-meal to 15 post-HIIT. Statistical differences were measured using RMANOVA.

Variable	Time Point	Oatmeal	Control	RMANOVA ^b^ (4 × 2)
ROS (μM/min)	Pre-meal	0.70 ± 0.1	0.75 ± 0.15	*p* < 0.05
	Pre-HIIT	0.69 ± 0.12	0.71 ± 0.12	
	Post-HIIT	0.78 ± 0.13 ^a^	0.94 ± 0.20	
	15 post-HIIT	0.72 ± 0.15	0.71 ± 0.11	
Glucose (mmol/L)	Pre-meal	5.18 ± 0.26	5.19 ± 0.43	*p* < 0.05
	Pre-HIIT	5.60 ± 0.65 ^a^	4.69 ± 0.34	
	Post-HIIT	4.80 ± 0.48 ^a^	5.33 ± 0.46	
	15 post-HIIT	5.4 3± 0.42	5.40 ± 0.49	
Lactate (mmol/L)	Pre-meal	1.31 ± 0.48	1.26 ± 0.36	*p* = 0.579
	Pre-HIIT	2.13 ± 0.94 ^a^	1.61 ± 0.38	
	Post-HIIT	5.18 ± 1.72	4.74 ± 2.04	
	15 post-HIIT	2.96 ± 1.19	3.13 ±1.30	

^a^ Significantly different from the control group at each time point following the independent *t*-test. ^b^ RMANOVA (4 × 2): four (time) × two (condition) repeated measures ANOVA. HIIT: high-intensity interval training and ROS: reactive oxygen species.

**Table 4:** Changes in the Borg Scale in the oatmeal group and control group from pre-meal to 15 post-HIIT. Statistical differences were measured using RMANOVA.

Variable	Time Point	Oatmeal	Control	RMANOVA ^a^ (4 × 2)
Borg	Pre-meal	6.88 ± 1.41	6.06 ± 0.25	*p* = 0.987
	Pre-HIIT	6.88 ± 1.26	6.31 ± 0.60	
	Post-HIIT	16.00 ± 1.41	15.25 ± 2.32	
	15 post-HIIT	8.19 ± 2.10	7.25 ± 1.81	

^a^ RMANOVA (4 × 2): four (time) × two (condition) repeated measures ANOVA.

## Data Availability

All datasets generated for this study are included in the article.
